# Effects of γ-PGA application on soil physical and chemical properties, rhizosphere microbial community structure and metabolic function of urban abandoned land

**DOI:** 10.3389/fmicb.2025.1534505

**Published:** 2025-05-30

**Authors:** Tu Shiheng, Ge Fanglan, Xu Yong, Sun Qingyi, Xie Fugui, Ren Yao, Du Juan, Chu Can, Xu Boyuan, Li Wei

**Affiliations:** ^1^College of Life Sciences, Sichuan Normal University, Chengdu, China; ^2^Key Laboratory of the Evaluation and Monitoring of Southwest Land Resources (Ministry of Education), Sichuan Normal University, Chengdu, China; ^3^Chengdu Zenith Chenxi Biotechnology Development Co., Ltd., Chengdu, China; ^4^Chengdu Jinguancheng Flower and Tree Horticulture Co., Ltd., Chengdu, China

**Keywords:** urban abandoned land, reclamation, γ-polyglutamic acid, soil properties, microbial community, metabolomics analysis

## Abstract

**Introduction:**

China’s rapid urbanization has led to the conversion of extensive farmland on urban fringes into non-grain uses, exacerbating the scarcity of arable land resources. Reclaiming these abandoned or underutilized areas presents a viable solution. However, many of these lands are contaminated with construction debris and have uneven soil quality, rendering them unsuitable for crop cultivation. This study aims to investigate the effects of γ-polyglutamic acid (γ-PGA) on improving such soils.

**Methods:**

A 6-month field experiment was conducted on green spaces with mixed construction waste in Chengdu’s urban ring. The study analyzed the impact of γ-PGA on soil bacterial communities, metabolites, and physicochemical properties during different wheat growth stages, namely tillering, jointing, flowering, and maturity.

**Results:**

γ-PGA significantly increased soil organic matter, total nitrogen, nitrate nitrogen, and alkali-hydrolyzable nitrogen. It also boosted enzyme activities such as urease, sucrase, and alkaline phosphatase. The soil mechanical structure improved, with increases in clay, sand, and macroaggregates. As wheat grew, the fractal dimensions of soil volume and infiltration performance increased, while bulk density decreased, indicating enhanced water retention and gas exchange. Beneficial microorganisms like Actinobacteria and Devosia increased in abundance, promoting soil fertility. Metabolomics analysis revealed that γ-PGA enriched pathways involved in carbohydrate digestion, starch metabolism, and nucleotide processes, creating a more favorable environment for plant growth.

**Discussion:**

This research underscores the crucial role of γ-PGA in soil restoration and fertility enhancement. The findings provide valuable insights for reclaiming non - grain farmland, offering a potential solution to the challenge of arable land shortage caused by urbanization. The study’s results contribute to the existing knowledge on soil improvement techniques and have practical implications for sustainable agricultural development in urbanized regions. However, further research could explore the long-term effects of γ-PGA application and its applicability in different soil types and environmental conditions.

## 1 Introduction

With the advancement of urban development and industrial innovation, many countries are grappling with large-scale environmental pollution and the challenge of redeveloping brownfields caused by urban construction activities. Over the past four decades, China’s urbanization has accelerated rapidly, with urban areas expanding at an average annual rate of 7.9% (Yu et al., 2022). Chengdu, the economic hub of southwestern China, exemplifies this urbanization trend. Between 1996 and 2016, the city’s rapid economic growth fueled continuous urban expansion, with its area increasing fourfold to 4,248 square kilometers (Luo et al., 2021; Zhao et al., 2024). According to recent land survey data, cultivated land in the Chengdu Plain has decreased by 40% over the past decade. This rapid urbanization and economic growth have also resulted in large amounts of abandoned or underutilized land, often temporarily used for construction waste disposal or converted into green spaces (Wu et al., 2022).

In 2020, Chengdu launched a plan to restore 100,000 mu of farmland within its “Ring Ecological Park,” aiming to align with national food security strategies and promote high-quality agricultural development. This initiative seeks to curb the conversion of farmland for non-food purposes and ensure stable grain production. Construction backfill areas and former landscaping spaces have been identified as potential farmland sites. However, field surveys indicate that these soils are of poor quality, containing mixed backfill materials with high gravel content and low nutrient levels, which limits their suitability for crop cultivation (Luo et al., 2021; Zhao et al., 2024). Therefore, finding effective methods to improve the structure and fertility of these reclaimed soils is essential.

Incorporating organic materials, such as crop residues, animal manure, and green manure, has proven effective in enhancing soil organic matter, improving structure, and boosting water and nutrient retention. Additionally, biological agents, such as earthworms and mycorrhizal fungi, can facilitate nutrient cycling and improve soil structure (Van Groenigen et al., 2019; Li et al., 2024). However, despite their benefits, these approaches carry potential risks, including heavy metal contamination, salt accumulation, and pH imbalances (Van Groenigen et al., 2019; Aparicio et al., 2022).

Poly-γ-glutamic acid (γ-PGA), a multifunctional biopolymer, offers great potential in agriculture due to its excellent water solubility, biodegradability, and environmental friendliness (Wang L. et al., 2022; Hu et al., 2023). Research by Yin et al. (2018) demonstrated that γ-PGA fermentation broth significantly increased the population of soil bacteria and fungi, promoting maize seedling growth. Furthermore, γ-PGA has shown potential in enhancing crop drought resistance (Nair and Dharne, 2023). Guo et al. (2023) reported that applying γ-PGA not only improved crop yields and quality but also enhanced the soil microenvironment (Bai et al., 2022; Guo et al., 2023). A two-year field experiment conducted by Liang and Shi (2021) in northwestern desert soils revealed that γ-PGA application significantly increased soil moisture retention, aggregate stability, and the yield and nutrient uptake efficiency of cotton. These studies highlight the potential of γ-PGA to improve soil structure, enhance water and nutrient use efficiency, and promote sustainable agricultural development. However, most previous studies were conducted in greenhouses or controlled environments, with limited research focusing on γ-PGA’s effects in open-field conditions. This study hypothesizes that the addition of γ-PGA to reclaimed urban green space soils will promote the formation of large soil aggregates, improve the soil’s physical and chemical properties, and increase crop yield and water-nutrient use efficiency in field conditions.

Currently, no studies have specifically investigated the application of γ-PGA in soils mixed with construction waste during green space reclamation. To address this gap, we conducted a six-month field experiment to systematically analyze the dynamic changes in soil physical and chemical properties, microbial communities, and metabolic functions following γ-PGA application. Additionally, this study explores the correlation between microbial activity and soil metabolite synthesis. The findings aim to provide scientific evidence for soil improvement and reclamation of urban wastelands and offer valuable insights for future efforts to enhance crop yield and quality.

## 2 Materials and methods

### 2.1 Research location and field experimental design

**Figure 1:**
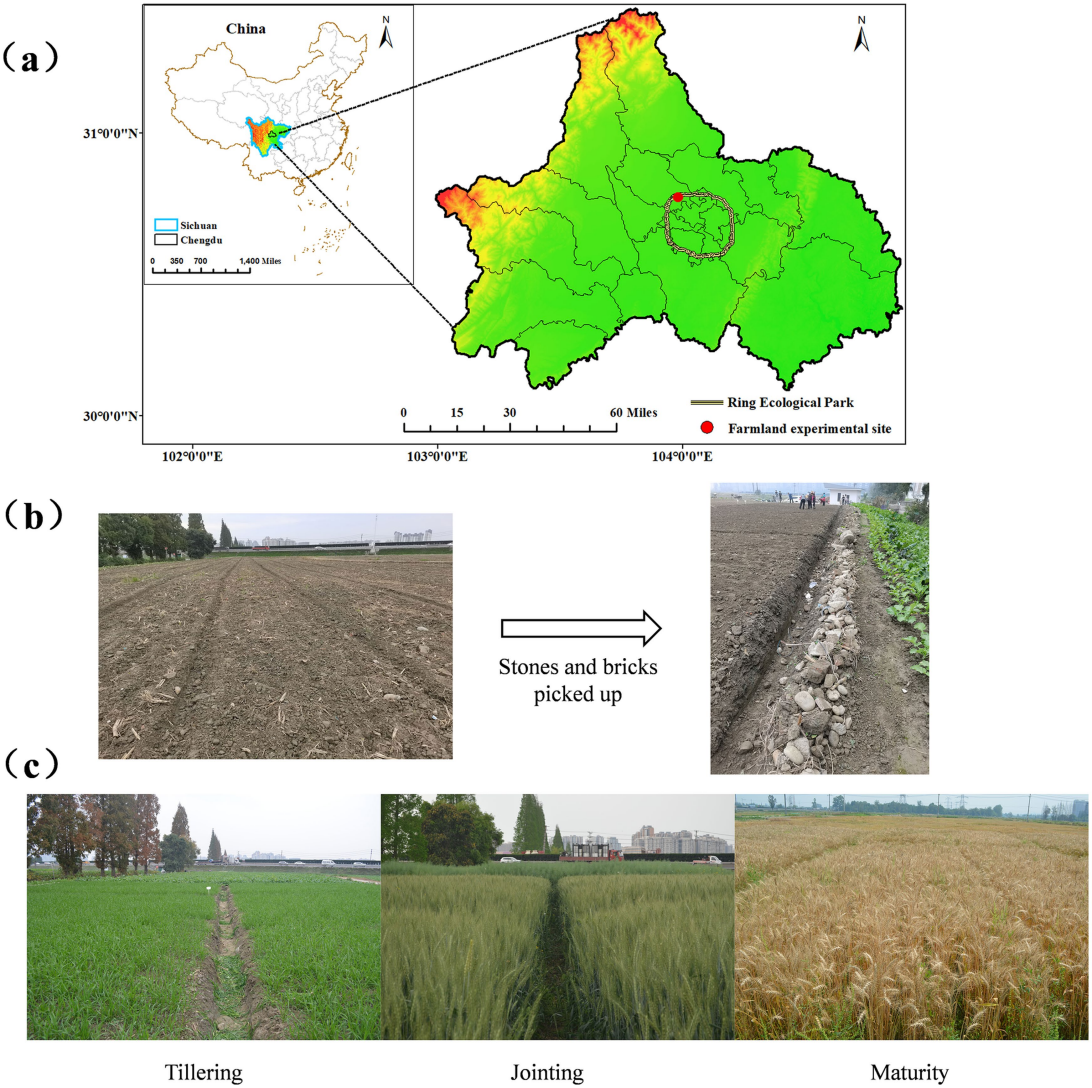
**(a)** Farmland experimental site; **(b)** Image of wheat experimental fields before the start; **(c)** Image of wheat experimental fields tillering, jointing and maturity (respectively).

The wheat variety used in this study was “Chuanmai 104.” The field experiment was conducted from October 29, 2023, to May 6, 2024, at a farmland site along the Urban Greenway on Datian Road, Pidu District, Chengdu, Sichuan, China (30°77′56″N, 103°97′31″E) (Figure 1a). The site was previously designated as green space, characterized by low soil moisture, a dry texture, fine particles, and an abundance of stones and bricks (Figure 1b). According to the USDA classification system, the soil type was identified as silty, with 12.14% clay, 85.12% silt, and 2.74% sand.

The baseline soil nutrient content included: organic matter (12.6 g/kg), total nitrogen (210 mg/kg), nitrate nitrogen (24.84 mg/kg), ammonium nitrogen (1.78 mg/kg), available nitrogen (21 mg/kg), available phosphorus (25.31 mg/kg), and exchangeable potassium (59.95 mg/kg). The soil pH was 6.83. The γ-PGA used in this study was purified from *Bacillus subtilis* SCP010-1 fermentation broth, with a molecular weight of approximately 1.1 million Da (Hong et al., 2024). Two experimental groups (B1 and B2) were established, with each treatment group consisting of four replicates. Each replicate corresponds to one plot covering an area of 83.33 m^2^, resulting in a total of eight plots, which were randomly arranged. Control group (B1): Fertilizer only. Experimental group (B2): Fertilizer combined with 0.4% γ-PGA powder (mixed with the fertilizer).

The field was manually cleared of stones, bricks, and plastic waste. A rotary tiller was used to incorporate either the base fertilizer or γ-PGA powder to a depth of 40 cm, ensure even distribution of fertilizer in the root zone. Wheat seeds were sown with a row spacing of 20 cm at a density of 3.5 million plants per hectare. Nitrogen fertilizer was applied in three stages using urea (46% nitrogen): 60% as base fertilizer, 20% at the tillering stage, and 20% at the jointing stage. Phosphorus and potassium were applied using calcium superphosphate (52% P₂O₅) and potassium sulfate (50% K₂O) as base fertilizers.

During jointing and flowering stages, accurately weigh γ- PGA powder, completely dissolve it in water, and the γ- PGA solution is evenly sprayed on the soil within 10 cm of the wheat roots in B2 group (300 g/mu each time) with a sprayer. Soil samples were collected at four key growth stages of wheat: tillering (T), jointing (J), flowering (F), and maturity (M).

### 2.2 Rhizosphere soil sampling

When more than 50% of the wheat plants reached the tillering, jointing, flowering, and maturity stages, samples were collected following standard protocols (Miller et al., 2003). The wheat development timeline was as follows: sowing: Oct 29, tillering: Nov 25–Dec 28, jointing: Feb 27–Mar 26, flowering: Apr 2–Apr 15, maturity: May 6 (Figure 1c).

Soil samples were collected on Dec 19, Mar 11, Apr 12, and May 6. For each group, three biological replicates were randomly selected for analysis. Intact wheat plants were carefully excavated. After gently shaking off the loosely attached soil from the roots, the tightly bound rhizosphere soil adhering to the root surface was collected using a sterile spoon to prepare a sample for subsequent analysis. Samples were sealed in plastic containers, transported on dry ice to the laboratory, and divided into two parts: one part was stored at −80°C for microbial and soil metabolomics analysis, the other part was air-dried, cleaned of impurities, ground, and sieved through 20-mesh and 100-mesh screens for physicochemical analysis.

### 2.3 Soil property measurement

The particle size distribution was measured using a Malvern Mastersizer 3000 particle size analyzer (UK), following Liu et al. (2013). Soil infiltration properties were assessed using vertical soil columns (Guo et al., 2021): soil column height: 30 cm; inner diameter: 5 cm, mariotte bottle height: 50 cm; inner diameter: 5 cm, soil height: 24 cm; constant water head: 2 cm.

Soil physicochemical properties were analyzed following the procedures in *Soil Agrochemical Analysis* (Rukun, 1999). Bulk density was determined using the ring knife method. Soil pH was measured with a pH meter after passing air-dried soil through a 2 mm sieve. Organic matter was analyzed using the high-temperature external heating potassium dichromate method. Total nitrogen was measured via the Kjeldahl method with potassium permanganate and ferrous sulfate modification. Available nitrogen, nitrate nitrogen, and available potassium were determined using colorimetric, alkaline diffusion, and flame photometry methods, respectively. Enzymatic activities were measured using Solarbio reagent kits (China): urease and sucrase activities were determined directly, while alkaline phosphatase was assessed via micro-assay kits.

### 2.4 Non-targeted soil metabolomics detection and analysis

LC–MS/MS analysis was conducted with five biological replicates per group by Majorbio Biotech Co., Ltd. (Shanghai, China), following Hou et al. (2021) with slight modifications. Rhizosphere metabolites stored at −80°C were ultrasonically extracted in 1 mL of methanol (4:1, v/v) containing internal standards at 5°C for 30 min. After centrifugation (13,000 g, 4°C, 15 min), the supernatant was concentrated under nitrogen gas. The samples were then dissolved in 120 μL of acetonitrile (1:1, v/v) and subjected to LC–MS/MS analysis using a Thermo UHPLC-Q Exactive HF-X system with an ACQUITY BEH C18 column (100 mm × 2.1 mm, 1.7 μm; Waters, United States).

Data processing was performed with Progenesis QI 2.3 software (Nonlinear Dynamics, Waters, USA) for peak detection and alignment. Identified metabolites were annotated using the KEGG database.^1^ Supervised clustering and PLS-DA were employed to assess group differences. Differential metabolites (DEMs) were defined by VIP > 1.0 and *p* < 0.05 (*t*-test) and visualized using volcano plots and bubble charts for enriched pathways.

### 2.5 Soil microbial diversity analysis

Microbial DNA was extracted from homogenized soil samples using the E.Z.N.A.^®^ Soil DNA Kit (Omega Bio-tek, United States). DNA concentration was measured using QuantiFluor™-ST (Promega, Beijing). The V3-V4 region of the 16S rRNA gene was amplified with primers 338F and 806R. Sequencing libraries were prepared using the TruSeq^TM^ DNA Sample Prep Kit, and sequencing was performed on the Illumina MiSeq PE300 platform. The original sequencing sequence has been stored in NCBI (BioProject ID: PRJNA1176352).

OTU-based methods were used for bioinformatic analysis, and alpha diversity metrics (coverage, Chao1, and Shannon indices) were calculated. Beta diversity was analyzed using Bray-Curtis distances with PCoA and NNMDS plots. ANOVA followed by post-hoc tests identified significant differences in taxa abundance at the genus level.

### 2.6 Correlation analysis between microbes and differential metabolites

The correlations between microbial communities and metabolite expression were analyzed using Pearson correlation, visualized with the *corrplot* R package. The relationships between bacterial genus-level diversity and DEMs were explored to identify potential interactions.

### 2.7 Statistical analysis

Data were processed using Excel 2010 and SPSS 17.0. One-way ANOVA followed by least significant difference (LSD) tests was employed to assess statistical significance among groups.

## 3 Results and discussion

### 3.1 Effects of γ-PGA application on soil texture

#### 3.1.1 Influence of γ-PGA on soil mechanical composition and aggregate proportion

Soil mechanical composition refers to the particle components within soil, such as clay, silt, and sand. The ratios and combinations of these components define soil texture and significantly influence properties like drainage, aeration, water retention, and other physical characteristics. This study examined the effects of γ-PGA on soil mechanical composition at various stages of wheat growth, including tillering, jointing, flowering, and maturity.

**Figure 2:**
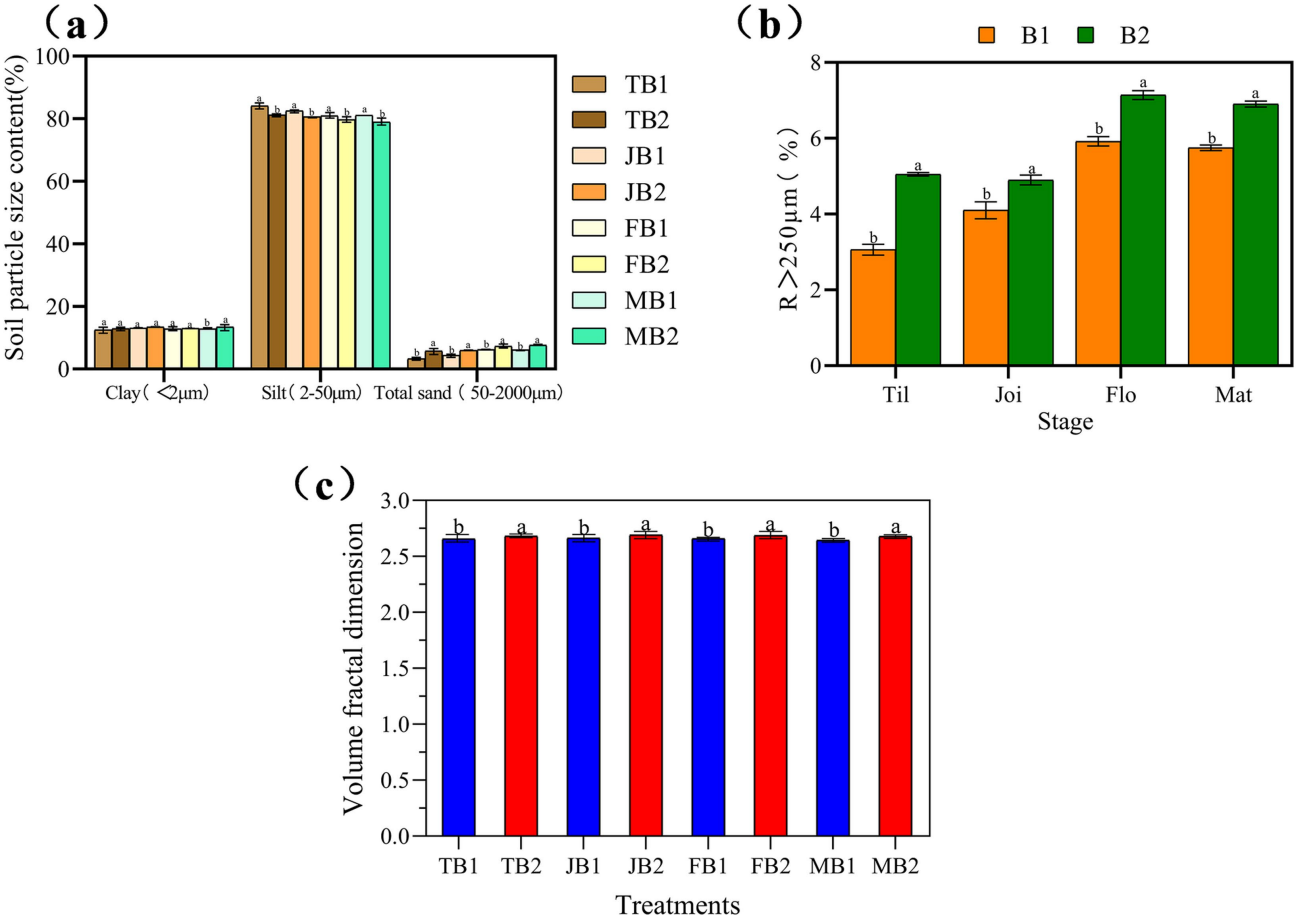
Effect of applying γ- PGA on soil texture and structure. **(a)** Soil texture; **(b)** Soil aggregates; **(c)** Volume classification dimension D of soil particles. Compared with B2 in the same period, ab: significant difference (*p* < 0.05), aa: insignificant difference. Considering that macroaggregates and microaggregates jointly account for 100% of the total, only the variation trend of macroaggregates is presented.

As illustrated in Figure 2a, the proportion of silt in the γ-PGA-treated group (B2) decreased by 2.91, 1.93, 1.27, and 2.11% across the four growth stages compared to the control group (B1). Conversely, clay content in the B2 group increased by 0.64% at the tillering stage, followed by increases of 0.36, 0.29, and 0.15% at the jointing, flowering, and maturity stages, respectively. Sand content also showed an upward trend, rising by 2.55, 1.64, 1.11, and 1.47% over the same periods.

Figure 2b further indicates that γ-PGA application significantly modified the soil aggregate structure. Specifically, the proportion of microaggregates (R < 250 μm) decreased, while macroaggregates (R > 250 μm) increased in the B2 group compared to the control. The largest increase in macroaggregate proportion was observed during the tillering stage, with a rise of 0.65-fold, followed by increases of 0.21-fold at flowering, and 0.20-fold and 0.19-fold at the jointing and maturity stages, respectively.

In summary, the application of γ-PGA fostered a gradual increase in macroaggregate formation throughout the wheat growth cycle. Concurrently, soil mechanical composition shifted, with reduced silt content and increased clay and sand content.

Alterations in soil mechanical composition play a fundamental role in modifying soil’s physical and chemical properties, impacting aspects such as fertility, water retention, thermal conductivity, and structural stability (Upadhyay and Raghubanshi, 2020). Lagomarsino et al. (2011) noted that soil conditioners like dolomitic limestone and compost significantly influenced mechanical composition. Our findings suggest that γ-PGA is an effective soil conditioner, reducing silt content while enhancing clay and sand proportions.

#### 3.1.2 Effect of γ-PGA on soil particle fractal dimension

The fractal dimension of soil particles is a key measure of soil structure complexity, indicating the distribution of particles and pore structures. A higher fractal dimension suggests more complex pore structures that enhance water retention and gas exchange, while a lower dimension indicates a simpler, more uniform distribution. Analyzing fractal dimensions provides valuable insights into soil structure, aeration, and water retention properties.

Compared to the B1 group, the γ-PGA-treated B2 group exhibited increases in fractal dimension at each of the four wheat growth stages: by 0.91, 1.06, 1.02, and 1.35% during tillering, jointing, flowering, and maturity, respectively (Figure 2c). This increase suggests that γ-PGA enhanced soil mechanical composition and improved soil structural complexity.

Higher soil fractal dimensions are associated with more complex pore morphology and more heterogeneous particle size distribution (Wang Y. et al., 2022). Similar results were reported by Hong et al. (2024) in pot experiments, where γ-PGA-treated soils displayed increased fractal dimensions across growth stages, aligning with the findings of this study.

### 3.2 Impact of γ-PGA on soil infiltration characteristics

**Figure 3:**
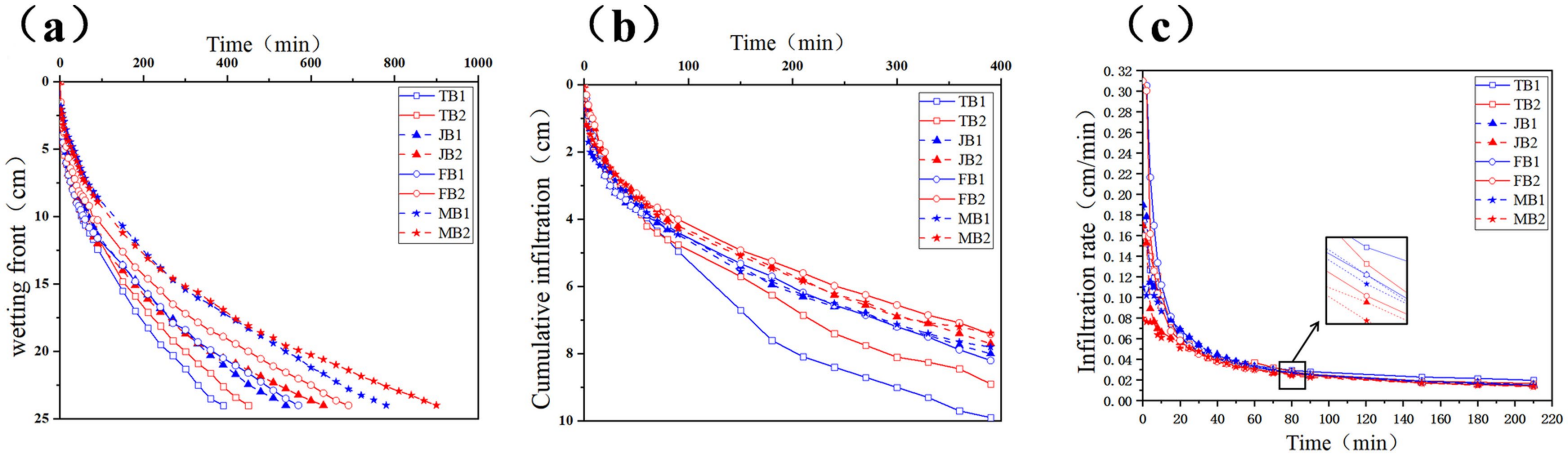
Effect of applying γ-PGA on soil infiltration characteristics. **(a)** Wetting front; **(b)** Cumulative infiltration; **(c)** Infiltration rate.

To investigate the effects of γ-PGA on soil infiltration, we conducted a one-dimensional column infiltration experiment, tracking the wetting front, cumulative infiltration, and infiltration rate over time (Figure 3). As shown in Figure 3a, with a wetting front of 24 cm, the infiltration rate in the B1 group was consistently faster than in the B2 group across all four growth stages. Specifically, the B1 group reached the wetting front 60 min earlier during the tillering stage, 90 min earlier during the jointing stage, and 120 min earlier during both the flowering and maturity stages.

As shown in Figure 3b, upon reaching a 24 cm wetting front in the B1 group, cumulative infiltration in the B2 group measured 8.9 cm (tillering), 7.7 cm (jointing), 7.42 cm (flowering), and 7.39 cm (maturity), representing reductions of 11.2, 3.9, 10.5, and 5.6%, respectively. As shown in Figure 3c, the infiltration rate stabilized after 80 min, with the B2 group demonstrating consistently lower rates across all stages: declines of 4.27% (tillering), 7.45% (jointing), 10.33% (flowering), and 5.8% (maturity).

Overall, γ-PGA application slowed the wetting front movement and decreased both the infiltration rate and cumulative infiltration, suggesting that γ-PGA improved soil water retention by reducing permeability. These findings align with Gao et al. (2024), who also observed enhanced water retention in shallow soils treated with γ-PGA.

### 3.3 Effect of γ-PGA on soil physicochemical properties

Soil samples were collected at four stages of wheat growth to compare the control group (B1) with the γ-PGA-treated group (B2), each with three biological replicates. As shown in Table 1, the bulk density of the B2 group was consistently lower than that of B1, with the most significant reduction of 14.17% observed during the maturity stage (MB2). Additionally, γ-PGA application increased soil pH, with the flowering stage (FB2) showing the largest increase of 2.62%. Organic matter content was also higher in the B2 group, with increases of 14.6, 18.85, 13.00, and 12.48% across the four growth stages.

**Table 1:** Effect of applying γ-PGA on soil properties.

Stage	Tilling		Jointing		Flowering		Maturity	
Treatment	TB1	TB2	JB1	JB2	FB1	FB2	MB1	MB2
Volumetric weight (g/cm^3^)	1.52 ± 0.01^a^	1.49 ± 0.02^b^	1.54 ± 0.03^a^	1.44 ± 0.01^b^	1.50 ± 0.02^a^	1.47 ± 0.02^b^	1.53 ± 0.01^a^	1.34 ± 0.01^b^
pH	7.23 ± 0.05^a^	7.31 ± 0.02^b^	7.27 ± 0.04^a^	7.42 ± 0.12^b^	7.26 ± 0.12^a^	7.45 ± 0.09^b^	7.38 ± 0.04^a^	7.48 ± 0.06^b^
Organic matter (g/kg)	13.14 ± 0.53^a^	15.06 ± 0.42^b^	13.00 ± 0.27^a^	15.45 ± 0.09^b^	15.93 ± 0.23^a^	18.00 ± 0.63^b^	15.87 ± 0.63^a^	17.85 ± 0.41^b^
Total nitrogen (mg/kg)	266.12 ± 9.15^a^	279.86 ± 5.11^b^	182.14 ± 18.75^a^	276.23 ± 24.35^b^	279.58 ± 4.86^a^	509.64 ± 7.45^b^	281.38 ± 13.42^a^	490.23 ± 8.14^b^
NO-N (mg/kg)	39.74 ± 0.97^a^	42.33 ± 0.52^b^	32.99 ± 2.10^a^	42.5 ± 1.21^b^	29.3 ± 0.29^a^	30.3 ± 0.35^b^	33.84 ± 0.18^a^	34.43 ± 0.30^b^
ASN (mg/kg)	21.40 ± 3.16^a^	35.20 ± 2.57^b^	24.50 ± 1.09^a^	33.60 ± 3.74^b^	30.10 ± 1.87^a^	35.00 ± 0.71^b^	22.40 ± 7.18^a^	42.00 ± 4.82^b^
Urease mg/(g*d)	3.27 ± 0.09^a^	3.66 ± 0.17^b^	4.22 ± 0.13^a^	4.55 ± 0.11^b^	5.79 ± 0.19^a^	6.72 ± 0.12^b^	5.87 ± 0.13^a^	6.64 ± 0.21^b^
S-SCmg/(g*d)	13.38 ± 0.37^a^	14.55 ± 0.21^b^	19.63 ± 1.39^a^	23.28 ± 0.54^b^	10.98 ± 0.84^a^	17.19 ± 1.12^b^	10.47 ± 0.63^a^	16.92 ± 0.34^b^
S-AKP (U/g)	9698.55 ± 571.48^a^	11094.99 ± 473.67^b^	10061.97 ± 956.49^a^	16847.39 ± 372.48^b^	10505.1 ± 1093.46^a^	17607.14 ± 428.98^b^	10247.45 ± 1462.73^a^	18788.27 ± 2109.3^b^
Olsen-K (mg/kg)	29.9 ± 0.27^a^	30.32 ± 0.18^b^	31.5 ± 0.08^a^	31.77 ± 0.25^b^	22.1 ± 0.79^a^	26.32 ± 1.32^b^	21.22 ± 0.43^a^	22.05 ± 0.27^b^

Data represent mean ± standard deviation (SD). LSD test was used to assess the differences. Different lowercase letters denote significant differences between the treatments (*p* < 0.05).

Regarding nitrogen content, γ-PGA notably increased total nitrogen, nitrate nitrogen, and alkali-hydrolyzable nitrogen levels. Enzyme activity also rose substantially, with urease activity increasing by 11.93, 7.82, 16.06, and 13.12%, and invertase activity rising by 8.74, 18.6, 56.56, and 61.6%. Alkaline phosphatase activity increased by 14.4, 67.44, 67.61, and 83.35% at each respective stage. Available potassium content was also significantly higher in the B2 group, with the greatest increase of 19.1% observed during the flowering stage (FB2).

These results indicate that γ-PGA application enhanced soil pH, organic matter, and nitrogen content while reducing bulk density. Fan et al. (2022) emphasized the importance of evaluating multiple indicators, including pH, bulk density, organic matter, and enzyme activity, to assess soil conditioner effectiveness. Our findings confirm that γ-PGA enhances soil fertility by improving mechanical composition and enzyme activity. Yin et al. (2018) also demonstrated that γ-PGA fermentation broth improved soil physicochemical properties in pot experiments, consistent with our observations. Recent studies further suggest that γ-PGA significantly reduces soil bulk density (Garbowski et al., 2023), promoting macroaggregate formation and improving aeration and water retention. These improvements foster a healthier soil environment, support plant growth, and contribute to sustainable development goals.

### 3.4 Analysis of soil microbial community structure

#### 3.4.1 Impact of γ-PGA application on soil bacterial community α-diversity

**Figure 4:**
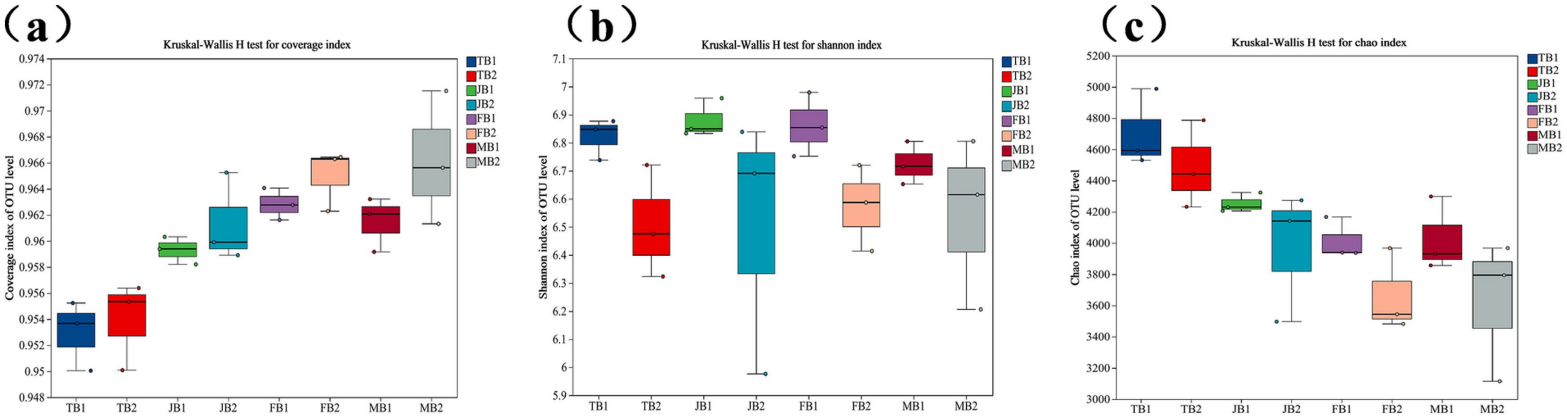
Effect of applying γ-PGA on the α diversity of soil bacterial communities. **(a)** Coverage; **(b)** Shannon; **(c)** Chao.

Soil samples from the B1 and B2 groups were collected at the tillering, jointing, flowering, and maturity stages of wheat, and the α-diversity of soil microbial communities was analyzed using high-throughput sequencing of the 16S rRNA gene. As shown in Figure 4, the species coverage (Goods coverage) for each sample exceeded 95%, indicating that the sequencing results accurately represent the microbial community composition within the samples. Compared to the control group, the γ-PGA-treated experimental group exhibited a slight increase in diversity indices.

Specifically, the Shannon and Chao1 indices for the experimental group showed a slight but statistically insignificant decline (*p* > 0.05), suggesting a trend toward reduced microbial diversity in the γ-PGA-treated soil, though the reduction was minor. This may be due to the expansion of a few dominant species outcompeting other species, resulting in an overall decrease in diversity. Similar findings were reported by Li et al. (2023), who observed that in rural soils with lower bacterial diversity, the Chao index remained high, and dominant bacteria, such as *Proteobacteria* and *Acidobacteria*, were more prevalent than in urban soils. This supports our findings, suggesting that a few dominant species may inhibit the growth of other microorganisms.

#### 3.4.2 Impact of γ-PGA application on soil bacterial community β-diversity

**Figure 5:**
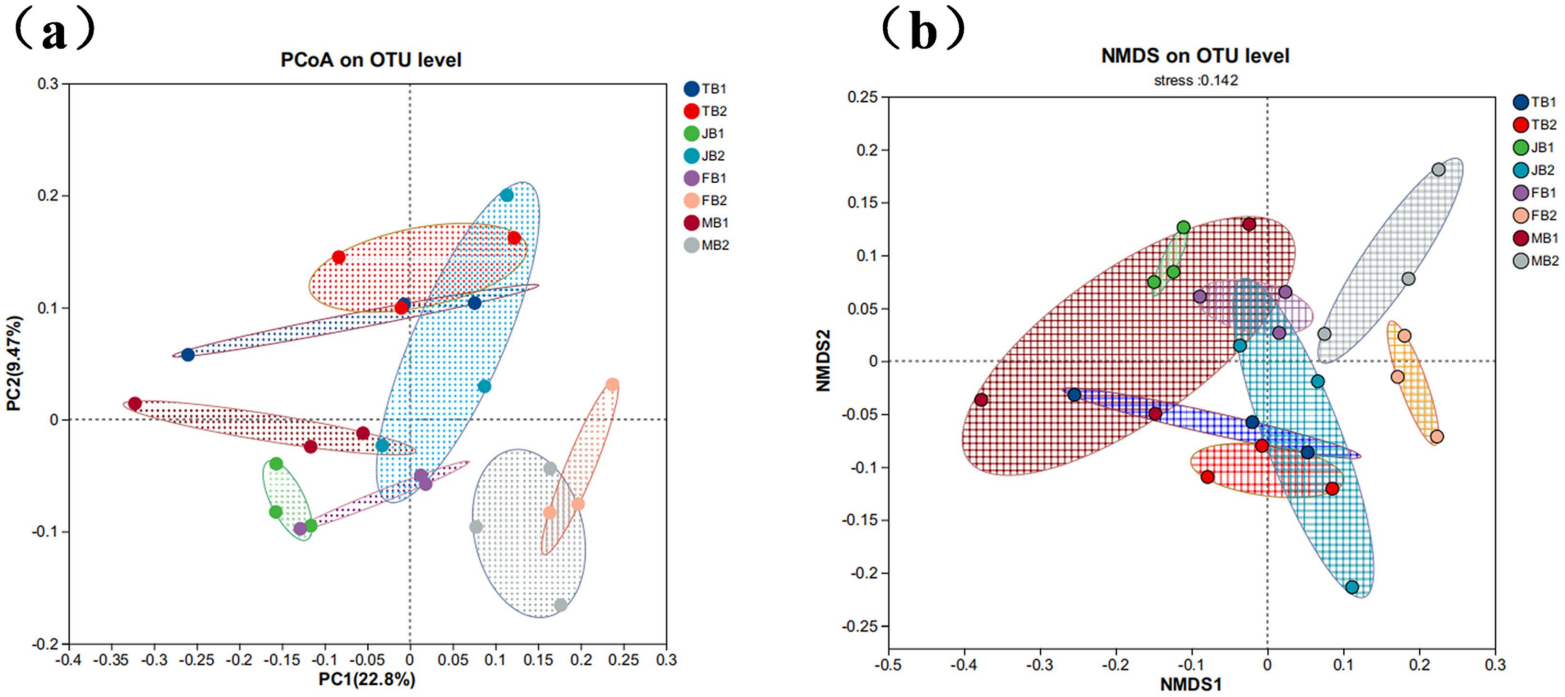
Effect of applying γ-PGA on the β diversity of soil bacterial communities. **(a)** PCoA analysis; **(b)** NMDS analysis (samples in different periods are presented in different colors, and the closer the sample points are, the more similar the OTU level composition between samples).

PCoA analysis based on Bray-Curtis distances revealed the effects of γ-PGA treatment on soil microbial community composition. As shown in Figure 5, the sample distribution of B1 and B2 group at four growth stages of wheat showed minimal overlap, indicating that the changes in soil microbial community composition were influenced by γ-PGA treatment (Figure 5a). The NMDS analysis results further confirmed this finding (Figure 5b). Although the statistical test did not reach a significant level, the comprehensive analysis results indicate γ-PGA treatment at different stages have a certain impact on soil microbial community structure.

#### 3.4.3 Impact of γ-PGA application on soil bacterial community composition

**Figure 6:**
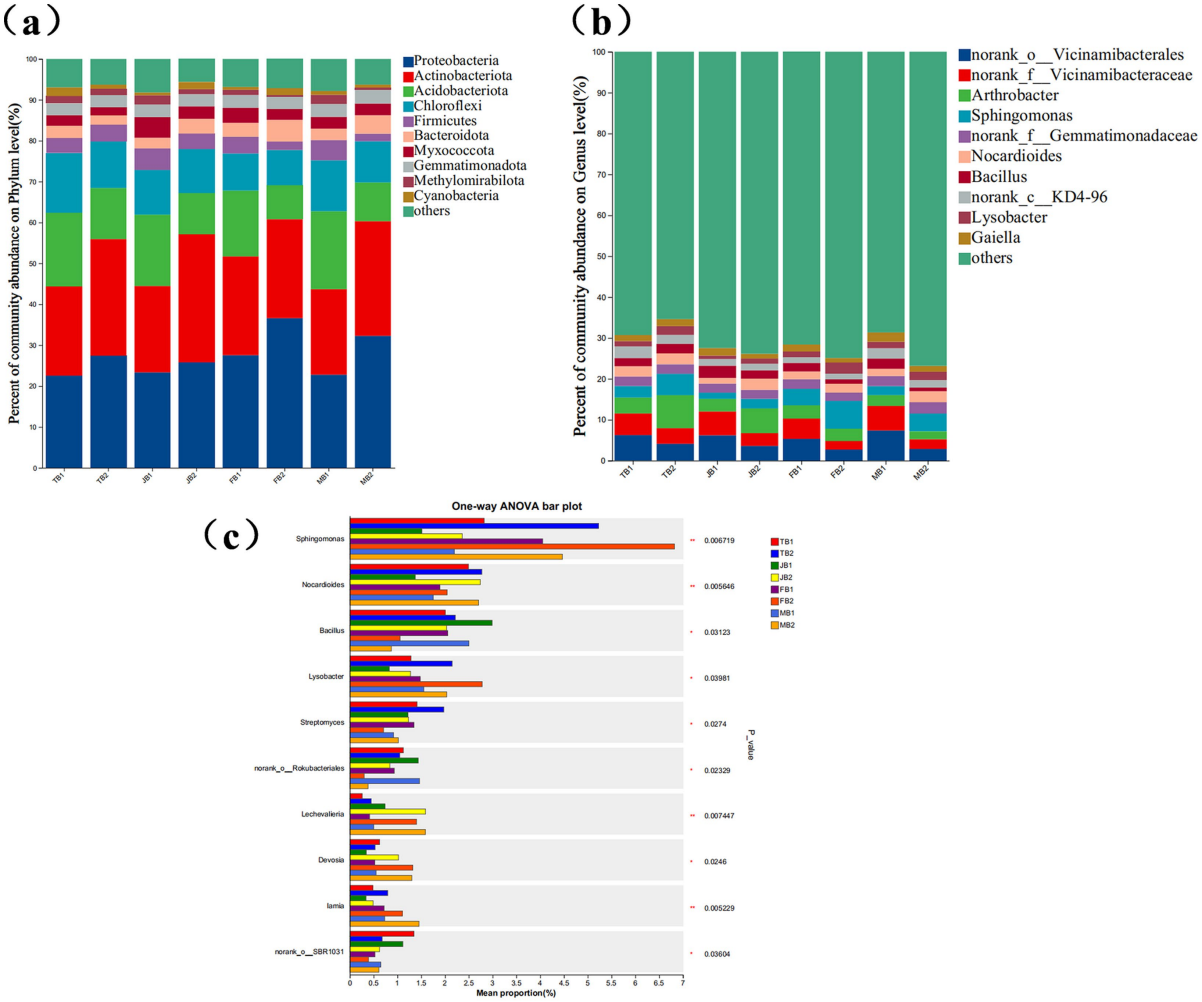
Effect of applying γ-PGA on soil bacterial community composition. **(a)** Stacked bar charts show the top 10 phyla with the highest relative abundance in each period; **(b)** Stacked bar charts show the top 10 genus with the highest relative abundance in each period; **(c)** Statistic the bacteria with significant differences in the genus level in different periods, display the differences in the average relative abundance of the same species in different periods with bar charts, and mark whether the differences are significant or not. It directly shows the significance of the difference of the same species among different groups. The Y-axis represents the species name at the genus level, and the X-axis represents the average relative abundance of the same genus in different periods; The number in the rightmost column is the value of *p*, and the asterisk represents the magnitude of significant *p*, *0.01 < *p* ≤ 0.05, **0.001 < *p* ≤ 0.01, ***p ≤ 0.001.

A total of 24 soil samples were collected across the four growth stages. In total, 46 phyla, 156 classes, 381 orders, 604 families, 1,179 genera, and 2,751 species were identified (see BioProject ID: PRJNA1176352). Proteobacteria and A*ctinobacteriota* were the primary dominant phyla, with total abundances ranging from 43.69 to 60.78%. Other prevalent phyla included *Acidobacteriota* (8.34–19.06%), *Chloroflexi* (8.62–14.56%), *Firmicutes* (1.85–5.28%), and *Bacteroidota* (2.17–5.31%) (Figure 6a).

At the genus level, six dominant genera (relative abundance > 1%) were identified: *norank_o__Vicinamibacterales* (2.67–7.38%), *norank_f__Vicinamibacteraceae* (2.11–6.00%), *Arthrobacter* (1.91–8.08%), *Sphingomonas* (1.51–6.82%), *norank_f__Gemmatimonadaceae* (2.09–2.65%), and *Nocardioides* (1.38–2.78%) (Figure 6b).

The application of γ-PGA notably influenced the relative abundance of dominant rhizosphere bacteria. Throughout various wheat growth stages, γ-PGA treatment increased the abundances of *Proteobacteria* and *Actinobacteriota*. In particular, at the maturity stage, the abundance of *Proteobacteria* increased by 9.5%, while *Actinobacteriota* increased by 10.07% during the jointing stage. At the genus level, the γ-PGA-treated group showed increased abundances of *Sphingomonas*, *Nocardioides*, *Lysobacter*, *Streptomyces*, *Lechevalieria*, *Devosia*, and *Lamia*. Among these, *Lechevalieria* and *Devosia* exhibited an average increase of 1.60-fold and 1.17-fold, respectively, while *Sphingomonas*, *Lamia*, *Lysobacter*, and *Nocardioides* increased by 0.65, 0.60, 0.43, and 1.1-fold, respectively (Figure 6c). These results suggest that although γ-PGA application did not alter the types of dominant bacteria, it did impact community diversity and structural proportions. The slight decrease in the Shannon and Chao1 indices may be attributed to the selective enrichment of beneficial microbial taxa, such as *Lechevalieria* and *Devosia*, by γ-PGA. This enrichment likely reduced the ecological niches available to other microorganisms, resulting in a non-significant overall decline in microbial diversity. This phenomenon is reasonable and suggests that γ-PGA promotes a more functionally optimized microbial community rather than broadly increasing microbial diversity. Overall, following γ-PGA application, the abundances of *Actinobacteriota*, *Lechevalieria*, *Devosia*, and *Lamia* increased progressively with wheat growth and eventually stabilized at high levels.

Drought resistance experiments by Yin et al. (2018) indicated that γ-PGA influences soil microbial community structure and diversity, with *Proteobacteria*, *Actinobacteriota*, and *Acidobacteria* becoming dominant communities that positively contribute to soil improvement. This aligns with our findings, suggesting that γ-PGA application enriches microorganisms such as *Proteobacteria*, *Actinobacteriota*, *Acidobacteria*, and *Chloroflexi*. Among them, members of *Actinobacteriota*, such as *Streptomyces*, are filamentous bacteria known for their antibiotic production and ability to degrade complex polymers like plant biomass (Hoskisson and van Wezel, 2019). Additionally, *Streptomyces* has shown environmental benefits in addressing plastic pollution (Verschoor et al., 2024), enhances drought tolerance in wheat seedlings (Yandigeri et al., 2012), and plays a key role in humus decomposition and formation (Buée et al., 2009). *Nocardioides* and *Lamia* are involved in nutrient cycling, pathogen suppression, enzyme production, and hormone secretion (Liu et al., 2023). Although *Lechevalieria* is less common, Zhiying et al. (2021) identified its potential for synthesizing abundant secondary metabolites that support plant growth (Liu et al., 2023; Zhang et al., 2023).

Members of *Proteobacteria* play essential roles in nitrogen fixation and polycyclic aromatic hydrocarbon degradation (García-Salamanca et al., 2013; Johnston-Monje et al., 2016). *Lysobacter* is valuable in biocontrol, producing multiple bioactive metabolites (Qian et al., 2013). *Sphingomonas* contributes to pollutant degradation and nitrogen cycling in soils, enhancing soil fertility and plant health (Aylward et al., 2013). Additionally, *Devosia* is advantageous for bioremediation in contaminated soils, utilizing its extensive oligopeptide permease (Opp) and dipeptide permease (Dpp) systems to absorb various peptides as carbon and nitrogen sources (Talwar et al., 2020). Furthermore, *Streptomyces* can secrete viscous substances like humus, promoting soil aggregate formation (Buée et al., 2009), while *Lechevalieria* contributes to soil structure stability through organic acid secretion and gas exchange (Fernández-Llamosas et al., 2021).

### 3.5 Metabolic differences in γ-PGA treated soil

**Figure 7:**
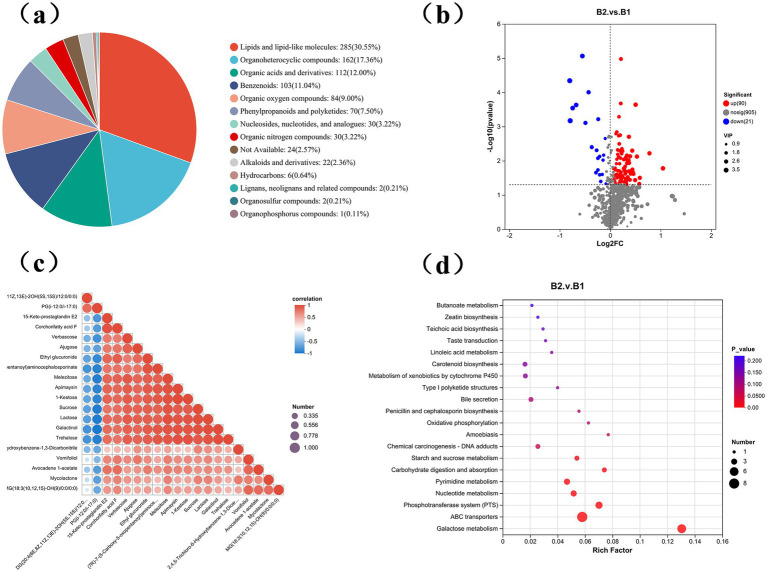
Metabolic differences in soil treated with γ-PGA. **(a)** Fan chart shows the composition of metabolites in soil samples; **(b)** Volcanic map of differential metabolites. The abscissa represents log2 (Fold Change) of metabolites in different groups, and the ordinate represents significance level [log10 (*p* value)]. Each point on the volcano map represents a metabolite, with significantly up-regulated metabolites represented by red dots and significantly down-regulated metabolites represented by blue dots. The size of each point represents a VIP value; **(c)** Triangular bubble thermogram shows the correlation between different metabolites, with different colors representing the magnitude of correlation coefficient, positive correlation coefficient and negative value indicating positive correlation and negative correlation, and the closer the absolute value is to 1, the higher the positive or negative correlation of metabolites; **(d)** The bubble diagram shows the enrichment pathway of differential metabolites, and the abscissa is the enrichment rate; The ordinate is KEGG pathway. In the figure, the size of bubbles represents the number of compound enriched in the metabolic concentration in this pathway, and the color of bubbles represents the size of different enrichment significance *p* values.

During the wheat jointing stage, soil samples were collected and analyzed using untargeted metabolomics to assess metabolite level changes between the B1 and B2 groups. A total of 995 metabolites were identified across 10 soil samples, categorized into 14 types (Figure 7a). These metabolites included lipids and lipid-like molecules (285, 30.55%), organic heterocyclic compounds (162, 17.36%), organic acids and derivatives (112, 12%), benzenoids (103, 11.04%), organic oxides (84, 9%), phenylpropanoids and polyketides (70, 7.50%), nucleotides and analogs (30, 3.22%), organic nitrogen compounds (30, 3.22%), unidentified metabolites (24, 2.57%), alkaloids and derivatives (22, 2.36%), hydrocarbons (6, 0.64%), lignans and related compounds (2, 0.21%), organic sulfur compounds (2, 0.21%), and organic phosphates (1, 0.11%).

Based on VIP values above 1.0 and *p*-values below 0.05, 958 metabolites were detected in the γ-PGA-treated group, with 90 upregulated and 21 downregulated, indicating an overall upward trend (Figure 7b). Visualization of the top 20 differential metabolites showed consistent upward trends, though the degree of change varied (Figure 7c).

**Figure 8:**
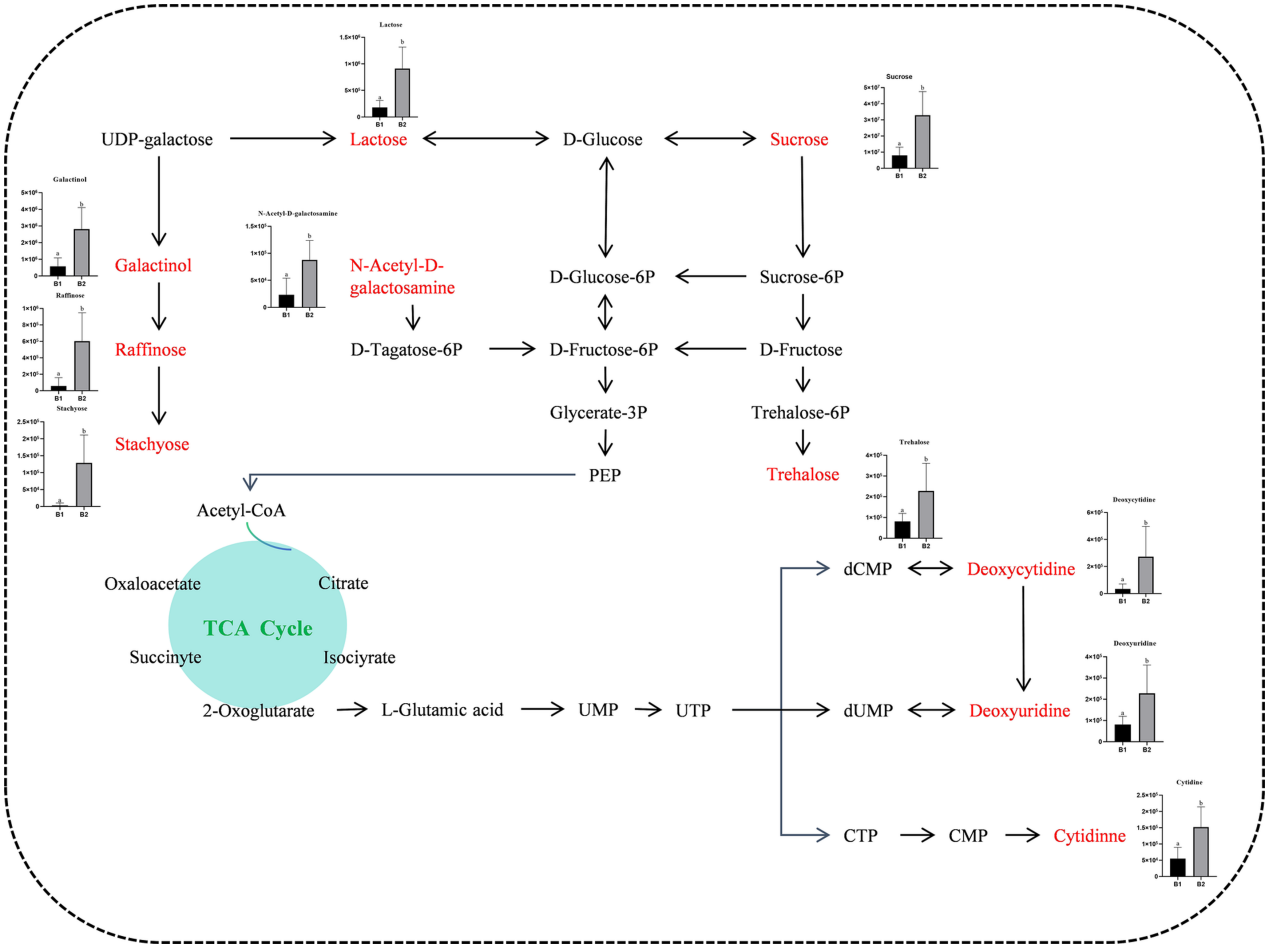
Metabolic pathway map of differentially up-regulated marker metabolites in soil treated with γ-PGA.

Seven significantly enriched metabolic pathways were identified using the KEGG database: carbohydrate digestion and absorption, starch and sucrose metabolism, nucleotide metabolism, pyrimidine metabolism, the phosphotransferase system, galactose metabolism, and the ABC transport system (Figure 7d). Furthermore, 11 upregulated differential metabolites participated in these enriched pathways, including sucrose, deoxycytidine, cytidine, N-acetyl-D-galactosamine, lactose, cellobiose, inositol galactoside, raffinose, deoxyuridine, trehalose, and stachyose. γ-PGA application significantly elevated soil metabolite levels, creating a new metabolic profile (Figure 8). These metabolite changes are likely to impact the composition of rhizosphere microorganisms, as they shape the microbial food web, regulate soil chemical properties, influence microbial gene expression, and serve as essential chemical signals in microbial interactions (Hu et al., 2018).

Our analysis of soil metabolites at the wheat jointing stage showed that lipids, lipid-like molecules, organic heterocyclic compounds, organic acids, and benzenoids accounted for approximately 71% of the metabolites. These metabolites provide essential nutrients for plant and soil microbial growth and are highly enriched in carbohydrate and nucleotide metabolism pathways. Additionally, eight significantly upregulated metabolites were enriched in the ABC transporter metabolic pathway, which is crucial for microbial nutrient uptake and utilization. ABC transporters are membrane-associated, energy-dependent transport proteins that move nutrients across cell membranes via ATP binding and hydrolysis (Greene et al., 2018). In high-carbohydrate environments, nutrients tend to form complexes with hydrophobic hydrocarbons, limiting microbial utilization (Obayori et al., 2015). γ-PGA treatment promotes microbial metabolic activity by enhancing nutrient transport.

Six differential metabolites were significantly enriched in the galactose metabolism pathway. For example, Vitamin C (AsA) not only provides plants with protection against drought, ozone, and UV radiation (Ali et al., 2019) but is also essential for microbial growth. AsA synthesis mainly depends on the L-galactose/D-mannose pathway, which is crucial for the health of both plant and soil microbial communities (Tao et al., 2020). Carbohydrate metabolic pathways, such as sucrose metabolism and the phosphotransferase system (PTS), play a vital role in enhancing soil fertility. PTS regulates complex carbon and nitrogen metabolism processes (Deutscher et al., 2006), thereby increasing soil fertility. Raffinose and stachyose are galactosyl sucrose derivatives belonging to the RFO family of oligosaccharides (Salvi et al., 2021), essential for transporting photosynthetic products in plants, especially within the Verbenaceae, Cucurbitaceae, and Lamiaceae families (Gangola and Ramadoss, 2018).

Trehalose, a disaccharide composed of two glucose molecules linked by an α-1-1 bond, has significant osmoprotective properties, helping maintain membrane lipid stability (Saddhe et al., 2021). Additionally, trehalose preserves protein structure and scavenges ROS (Zulfiqar et al., 2019), enhancing plant tolerance to abiotic stresses. The levels of sucrose, lactose, and trehalose were significantly elevated in γ-PGA-treated soil, potentially contributing to improved plant stress resistance.

In conclusion, γ-PGA application significantly altered the soil’s metabolic landscape by modulating the types and concentrations of various chemical metabolites. This not only improved the soil’s physical and chemical properties, creating a more favorable environment for plant growth, but also provided essential nutrients for microbial activity. By promoting microbial growth and reproduction, γ-PGA treatment enhances the diversity and functionality of soil microbial communities, further increasing soil fertility and plant resilience to stress.

### 3.6 Correlation analysis between microbial genera and differential metabolites

#### 3.6.1 Procrustes analysis

**Figure 9:**
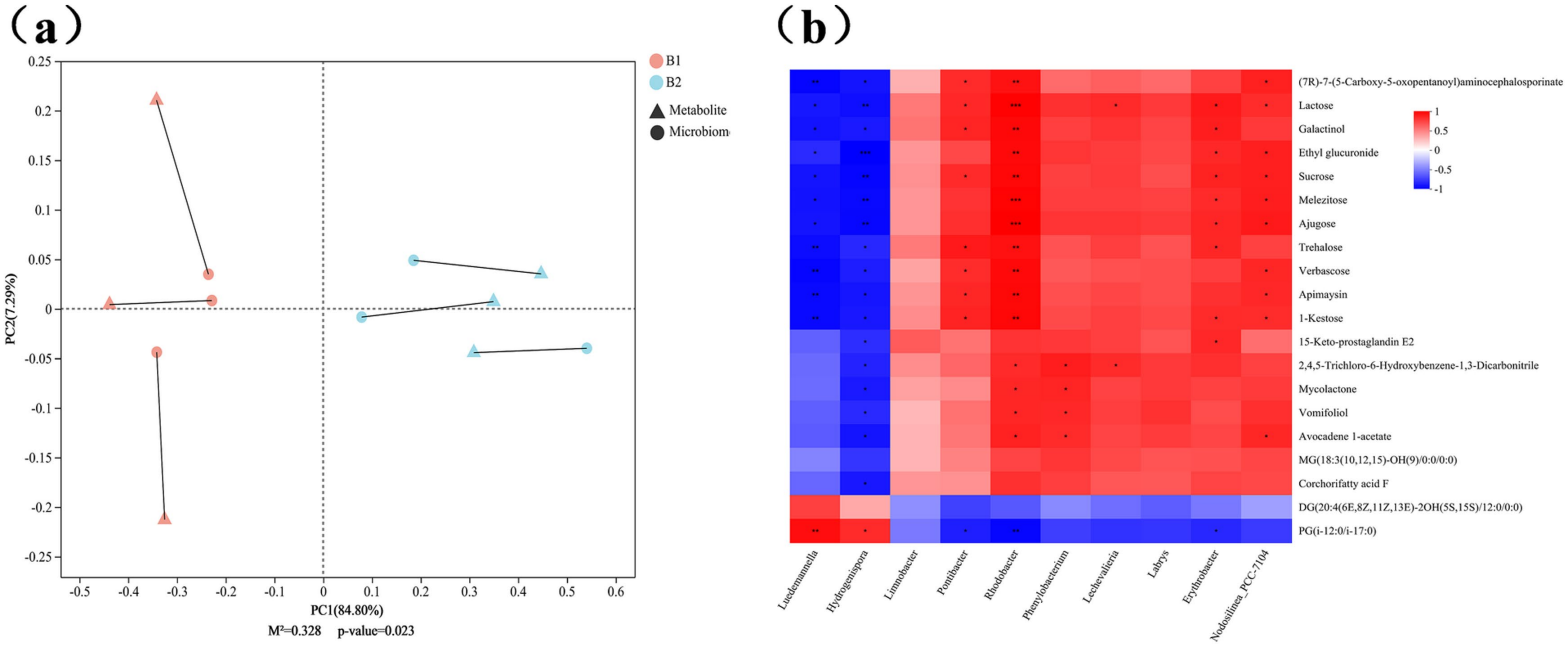
Correlation analysis between microbial genera and differential metabolites. **(a)** Procrustes analysis, in which different colors represent different groups of samples; Each line segment represents a sample, the solid dot at one end of the line segment represents the PCA ranking result of related (16S diversity sequencing or phenotype) data, and the other end of the line segment represents the ranking result of metabolite composition data obtained by metabonomics analysis of the same sample; The connection line represents the Procrustes residuals of two sorting configurations, which can evaluate the variation between them. The longer the connection line, the lower the consistency between the two data sets. **(b)** Thermal map of the correlation between different microorganisms and different metabolites, with the name of metabolites on the right side and the name of microorganism at the bottom. Each grid in the map represents the correlation between two attributes (metabolites and microorganisms), and different colors represent the magnitude of correlation coefficient between attributes. The asterisk represents the magnitude of significance *p*, *0.01 < *p* ≤ 0.05, **0.001 < *p* ≤ 0.01, ***p ≤ 0.001.

A correlation analysis between microbial genera and differential metabolites was conducted using data from the wheat jointing stage. Procrustes analysis, a multivariate statistical method, was used to assess the consistency of shape between different datasets. By matching corresponding points (coordinates) in the two datasets, Procrustes analysis applies translation, rotation, and scaling to one dataset to minimize the sum of squared deviations (M^2^) between the datasets. The *p*-value measures the significance of the correlation trend between microbial and metabolite expression levels. As shown in the Figure 9a, a *p*-value of 0.023 (*p* < 0.05) indicates a significantly consistent trend in microbial and metabolite expression levels across different treatment groups.

#### 3.6.2 Relationship between microbial genera and differential metabolites

Bacterial genera showing significant differences at the genus level were identified from the microbial diversity analysis, and correlation analysis with differential metabolites from the metabolomics data was performed using the Pearson correlation coefficient, with results displayed in a heatmap (Figure 9b). The results revealed strong correlations between most of the 10 microbial genera and 20 differential metabolites, suggesting that these microbes may play roles in soil metabolite formation. Further analysis identified potential associations between 11 key metabolites and specific bacterial genera. These metabolites include trehalose, verbascose, kestose, lactose, raffinose, sucrose, melezitose, myo-inositol galactoside, ethyl glucuronide, palmitic aldehyde, and (7R)-7-(5-carboxy-5-oxopentanoyl) aminocephalosporin, which were significantly correlated with four genera: *Pontibacter*, *Rhodobacter*, *Nodosilinea_PCC-7104*, and *Erythrobacter* (Figure 9b).

Previous studies have shown that soil microbial communities are major drivers of soil metabolic changes (Ren et al., 2022), while bioactive metabolites, in turn, influence microbial diversity (Bi et al., 2022). This study further analyzed associations between differential metabolites and microbial genera in the B1 (CK) and B2 (γ-PGA) groups. The results indicated that key metabolites such as kestose, sucrose, and lactose were closely related to *Pontibacter*, *Rhodobacter*, *Nodosilinea_PCC-7104*, and *Erythrobacter*. Literature suggests that members of the *Pontibacter* genus have the potential to metabolize polysaccharides (Ma et al., 2023) and can utilize sugars such as fructose and lactose as carbon sources (Dastager et al., 2010). Additionally, most members of the *Rhodobacter* and *Erythrobacter* genera contain chlorophyll a and can perform photosynthesis, using simple sugars as an energy source (Tonon et al., 2014; Imhoff, 2015). Although studies on *Nodosilinea_PCC-7104* are limited, this genus is known to adapt to alkaline soils and can use TDN and NO₃^−^-N as primary nutrient and energy sources (Song et al., 2022; Zhang et al., 2022), consistent with the physicochemical findings of this study. However, further research is needed to clarify *Nodosilinea_PCC-7104*’s role in sugar metabolism. This study demonstrates that γ-PGA application modifies soil microbial community structure, enhancing interactions between specific beneficial bacteria and key metabolites, which play an important role in the soil ecosystem.

## 4 Conclusion

This field reclamation study investigated the effects of γ-PGA on soil properties in green land mixed with construction debris, examining soil mechanical composition, infiltration characteristics, basic physicochemical traits, and microbial structure. γ-PGA application across four wheat growth stages (tillering, jointing, flowering, and maturity) significantly altered soil composition, reducing silt while increasing clay, sand, and macroaggregates, thus enhancing soil aggregation and structural integrity. Additionally, γ-PGA improved the fractal dimension of soil particles across all stages, suggesting increased complexity in pore structure that favors water retention and gas exchange. γ-PGA-treated soils showed lower infiltration rates and cumulative infiltration than controls, highlighting γ-PGA’s role in enhancing soil water retention. Furthermore, γ-PGA application lowered soil bulk density, raised pH and organic matter content, and boosted nitrogen levels and enzyme activities, demonstrating its effectiveness in enhancing soil fertility. Microbial community analysis revealed that γ-PGA influenced both α- and β-diversity of soil bacterial communities, with marked differences in composition and structure throughout wheat growth stages. In sum, γ-PGA functions as a robust soil conditioner, optimizing soil’s physical and chemical characteristics and fostering microbial diversity. Its application significantly improves soil fertility and supports sustainable agricultural development by creating a healthier, more resilient soil environment. It should be noted that this study involved only a six-month field trial, which is relatively short. Long-term trials across multiple crops and over several years are still needed to further verify the applicability and scalability of γ-PGA. Therefore, the current research can be regarded as a preliminary exploration, and its various effects require more in-depth validation.

## Data Availability

The datasets presented in this study can be found in online repositories. The names of the repository/repositories and accession number(s) can be found at: https://www.ncbi.nlm.nih.gov/, PRJNA1176352.

## Footnotes

^1^ https://www.kegg.jp/
